# Can lipid indices aid in predicting diabetic kidney disease? Findings from a cross-sectional, matched case-control study

**DOI:** 10.1371/journal.pone.0331756

**Published:** 2025-10-07

**Authors:** Amirhossein Yadegar, Fatemeh Mohammadi, Fatemeh Heydarzadeh, Kiavash Mokhtarpour, Sepideh Yadegar, Rana Hashemi, Seyed Ali Nabipoorashrafi, Soghra Rabizadeh, Alireza Esteghamati, Manouchehr Nakhjavani

**Affiliations:** Endocrinology and Metabolism Research Center (EMRC), Vali-Asr Hospital, Tehran University of Medical Sciences, Tehran, Iran; University of Pisa, ITALY

## Abstract

**Background:**

This study explored the association between lipid indices, including AC, TG/HDL-C ratio, AIP, and LCI, and diabetic kidney disease (DKD).

**Methods:**

A cross-sectional, matched case-control study was conducted involving patients with type 2 diabetes (T2D), divided into two groups based on the presence of DKD. The groups were matched for age and duration of diabetes. The association between lipid indices and DKD was assessed using RCS, multivariable logistic regression, and ROC curve analysis.

**Results:**

The study included 2940 individuals with T2D, with 1470 in each group. A nonlinear association was observed between all lipid indices and the presence of DKD. These lipid indices demonstrated relatively high predictive ability for DKD, with all AUC values higher than 0.707. The AIP and TG/HDL-C ratio had the highest AUCs of 0.717 and 0.713, respectively. Both indices also exhibited the highest sensitivity at 68%, while LCI showed the highest specificity at 79%. After adjusting for potential confounders, all lipid indices were significantly associated with DKD in the multivariable logistic regression analysis. Non-linear associations were found between lipid indices and components of DKD. All lipid indices demonstrated significant relationships with uACR ≥ 30 mg/g, whereas only AIP showed a significant association with eGFR < 60 mL/min/1.73m^2^. According to the ROC curve analysis, AIP was the most effective at identifying reduced eGFR (AUC = 0.676 [0.637–0.712]), and LCI was the best performer for detecting elevated uACR (AUC = 0.741 [0.701–0.783]).

**Conclusions:**

Lipid indices may serve as valuable, non-invasive tools for the early detection of DKD, potentially leading to effective diabetes management and reducing the burden of DKD.

## Introduction

Diabetic kidney disease (DKD) affects approximately 25% to 40% of patients with type 2 diabetes (T2D) and is a leading cause of chronic kidney disease (CKD), especially in developed countries [1,2]. DKD not only contributes substantially to the development of end-stage renal disease (ESRD) but is also linked to an increased risk of cardiovascular disease (CVD), diabetic foot complications, and overall mortality [1,3].

The progression of DKD is multifactorial, with insulin resistance and ectopic lipid accumulation in the glomeruli playing pivotal roles in podocyte dysfunction and CKD development [4–7]. Moreover, DKD is associated with abnormal lipid metabolism due to decreased kidney function, characterized by elevated total cholesterol (TC) and triglycerides (TG), along with decreased high-density lipoprotein cholesterol (HDL-C) levels [8–10]. Lipid-lowering therapies have demonstrated the ability to delay the DKD progression [8].

Timely identification of DKD is crucial for early intervention, targeted therapy, and preventing the progression of DKD to ESRD [2,11]. Although the measurement of 24-hour urinary albumin is considered as one of the most sensitive markers of early glomerular damage, its accuracy can be compromised by various factors, which limits its practical use [12]. Therefore, there is a need for simple, cost-effective, and reliable markers for the early detection of DKD in clinical practice [12].

Recent studies indicate that novel lipid indices, such as the atherogenic index of plasma (AIP), atherogenic coefficient (AC), and Castelli risk index (CRI) I and II, are more effective in identifying the prevalence and severity of diabetic complications than traditional lipid profile measures, including TG, low-density lipoprotein cholesterol (LDL-C), and HDL-C [13]. These indices can be easily calculated in clinical settings and provide a more comprehensive assessment of dyslipidemia compared to individual lipid profile components [14]. For instance, the AIP offers a better understanding of the pathogenesis and specific nature of dyslipidemia than evaluating high TG and low HDL-C levels alone [15–18]. Studies have shown that elevated AIP is independently associated with an increased risk of DKD [11]. Other indices, including CRI and AC, have been recognized as independent risk factors for coronary artery disease (CAD) [19]. The triglycerides/high-density lipoprotein cholesterol (TG/HDL-C) ratio has been proposed as a screening tool for conditions such as insulin resistance, metabolic syndrome, T2D, CKD, and CVD [20–23]. While most studies have focused on the association and utility of lipid indices in predicting macrovascular complications in diabetes, there is a paucity of research exploring the relationship between these indices and DKD.

This study aimed to investigate the association between novel lipid indices (AC, TG/HDL-C ratio, AIP, and lipoprotein combine index (LCI)) and DKD, and to assess their predictive value for identifying DKD in patients with T2D.

## Materials and methods

### Study design and population

This cross-sectional, matched case-control study evaluated individuals with T2D referred to the diabetes clinic of a tertiary hospital affiliated with the Tehran University of Medical Sciences. Diabetes was diagnosed based on the criteria of the American Diabetes Association (ADA): fasting blood sugar (FBS)≥126 mg/dL, hemoglobin A1c (HbA1c)≥6.5%, 2-hour postprandial blood glucose (2hpp)≥200 mg/dL, or a random plasma glucose ≥200 mg/dL along with classic symptoms of hyperglycemia or hyperglycemic crisis [24]. Participants with thyroid disease, malignancies, liver cirrhosis, pregnancy, smoking, or those taking oral contraceptives or antioxidant supplements were excluded due to the potential influence of these conditions on lipid metabolism [25,26]. Participants were categorized into two groups based on the presence or absence of DKD, matched for age and diabetes duration using propensity score matching (PSM). The study population was relatively homogenous, comprising middle-class individuals with educational backgrounds ranging from middle to high school. The participants had healthcare access and insurance coverage. The data were accessed on June 20, 2024. All data were anonymized, and the authors did not have access to any information that could identify individual participants, either during or after data collection. The Research Ethics Committee of Tehran University of Medical Sciences approved the study. Prior to participation, all individuals provided written consent. The study adhered to the ethical standards outlined in the Declaration of Helsinki.

### Data collection and variables

Demographics, anthropometric measurements, and biochemical analyses were performed. Age, gender, and diabetes duration were recorded using a standardized questionnaire, along with a detailed medical and medication history. Anthropometric measurements, including blood pressure, waist circumference (WC), height, and weight, were determined by well-trained investigators. WC was measured by non-elastic tape at the middle point between the inferior lower borders of the rib cage and the iliac crest (rounded to the nearest 0.1 cm) [27]. Body weight was assessed using a digital scale (Tefal PP1100), with participants dressed in light clothing and barefoot (nearest 0.1 kg). Height was determined using a measuring tape while participants stood upright against a wall with their feet together (nearest 0.1 cm). Body mass index (BMI) was computed by dividing weight in kilograms by the square of height in meters. Blood pressure (systolic and diastolic) was measured using a calibrated Omron M7 digital sphygmomanometer (Hoofddorp, The Netherlands) and a suitably sized cuff on the right arm of participants who were seated and had a five-minute rest period. The average of two measurements taken ten minutes apart was recorded for systolic blood pressure (SBP) and diastolic blood pressure (DBP). During the assessments, participants were asked to sit with their right hands at heart level and palms facing up. Hypertension was considered positive in participants with SBP ≥ 140 mmHg, DBP ≥ 90 mmHg, or being on therapy for hypertension [28]. A venous blood sample was taken from each individual after 12 hours of fasting to measure FBS, HbA1c, creatinine, TG, TC, LDL-C, and HDL-C. Another venous blood sample was taken two hours after the beginning of an adequate meal to measure 2hpp. The glucose oxidase method was employed to measure FBS and 2hpp (Parsazmun Auto Analyzer, BT-3000(plus), Biotechnica). HbA1c was assessed through high-performance liquid chromatography (HPLC) using the DS5 instrument from DREW, England. TC, TG, LDL-C, HDL-C, and creatinine were quantified using enzymatic techniques (Parsazmun Auto Analyzer, BT-3000(plus), Biotechnica). Non-HDL-C was calculated from the subtraction of HDL-C from TC. The estimated glomerular filtration rate (eGFR) was determined using the Chronic Kidney Disease Epidemiology Collaboration equation (CKD-EPI) [29]. Patients were asked to gather both spot and 24-hour urine for albumin and creatinine level analysis. The spot urine sample was collected in the morning while fasting. In the case of 24-hour urine collection, boric acid-supplied containers were given to each participant to collect the urine for an entire 24-hour period starting at 7 am. The urinary albumin excretion (UAE) level was assessed using the latex turbidimetric immunoassay method with the DAKO package from Glostrop, Denmark. Urinary creatinine concentrations were analyzed using the Jaffe colorimetric assay and an automated system (Pars Azmun, Iran). The urine albumin-creatinine ratio (uACR) was calculated by dividing the urine albumin (mg/dL) by the urine creatinine (g/dL) using the spot urine sample. The lipid indices including AC, AIP, and LCI were calculated by the following formulas: AC = (TC − HDL-C)/HDL-C, AIP = log (TG/HDL-C), LCI = TC*TG*LDL-C/HDL-C.

### Medication use

According to glycemic level, either monotherapy or multiple drug therapy was prescribed. Monotherapy involves the use of oral anti-diabetic drugs (OAD) or insulin, while multiple drug therapy includes the use of two or more OADs or a combination of OADs and insulin. Regarding participants with insulin treatment, the insulin glargine or NPH was used along with mealtime insulin aspart or regular. The dyslipidemia medications were atorvastatin, rosuvastatin, and fibrates, with majority of the population prescribed atorvastatin because of its availability and cost-effectiveness. Overall, the medication compliance among the participants was consistent.

### Definition of DKD

DKD was defined as an eGFR below 60 mL/min/1.73m^2^, a uACR ≥ 30 mg/g, persistent albuminuria >300 mg/day, or a combination of them [30]. Patients with proteinuria or renal dysfunction unrelated to diabetes were excluded. The diagnosis of DKD was validated using the ICD-10 code E11.2.

### Statistical analysis

Data were analyzed using Python version 3.12 with NumPy version 1.26, Pandas version 2.1.4, Matplotlib version 3.8.1, Scikit-learn version 1.4.0, SciPy version 1.13.1 libraries [31–35], and SPSS version 22.0 (IBM Corporation, USA). Continuous variables were reported as mean ± standard deviation (SD) or median (Q1–Q3), while categorical variables were expressed as frequencies and percentages. Normality was assessed using the Kolmogorov-Smirnov test and visual inspection of P-P plots and histograms. Parametric (Student’s t-test) and non-parametric (Mann-Whitney U test) tests were used for group comparisons as appropriate. The chi-squared test was utilized to analyze categorical variables.

A restricted cubic spline (RCS) was utilized with four knots centered at the 5th, 35th, 65th, and 95th percentiles of various lipid indices to model the potential non-linear association between DKD and the values of lipid indices. The median of each index served as the reference point, with an odds ratio (OR) of 1, to which other values were compared. This study conducted a multivariable logistic regression analysis to assess the association between quartiles of various lipid indices and DKD. Initially, unadjusted analysis was performed, followed by adjustments for sex, hypertension, dyslipidemia drug, BMI, antihyperglycemic agents, and HbA1c, all of which showed significant differences between the groups. Results were reported as odds ratios (ORs) and 95% confidence intervals (CIs). A receiver operating characteristic (ROC) curve was used to estimate the predictive values of different lipid indices for the DKD, and the cut-offs were calculated using the maximum Youden index. The ROC curve was adjusted for sex, hypertension, dyslipidemia drug, BMI, antihyperglycemic agents, and HbA1c. The multicollinearity was assessed using the Variance Inflation Factor (VIF) and lipid indices with high multicollinearity (VIF > 5), including CRI-I and CRI-II, were excluded from the main analysis. Nevertheless, their results are mentioned in the supplementary material (S1 File). The same analytical models were also conducted separately for each component of the DKD definition, including eGFR < 60 mL/min/1.73m^2^ and uACR ≥ 30 mg/g. In this study, a p-value<0.05 was considered statistically significant.

## Results

### Baseline characteristics according to DKD status

In the current study, 2940 individuals were enrolled, 56.6% of whom were women. The mean age of the total population and both groups with and without DKD was 62 years. The median duration of diabetes was 10 and 9 years in participants with and without DKD, respectively. The average BMI level among participants with DKD was significantly higher by 29.1 kg/m^2^ over 28.1 kg/m^2^ (P-value<0.001). 56.7% of the DKD group had hypertension, which was significantly higher than the prevalence of hypertension in the group without DKD (45.1%) (P-value<0.001). Additionally, the SBP, DBP, WC, 2hpp, HbA1c, and creatinine levels had significantly higher values among the group with DKD (Table 1). The level of eGFR was significantly greater in the group without DKD than in the DKD group by 80.3 over 62.6 ml/min/1.73m^2^ (P-value<0.001). Patients with DKD had higher TG, TC, and non-HDL-C levels (P-value<0.001 for TG and non-HDL-C, P-value = 0.031 for TC). HDL-C was significantly higher among individuals without DKD (P-value<0.001). No significant differences were found between the two groups regarding FBS and LDL-C (Table 1). As Table 2 illustrates, all of the lipid indices including AC, TG/HDL-C ratio, LCI, and AIP were significantly higher in the group with DKD (P-value<0.001).

**Table 1:** Baseline characteristics of the study population by diabetic kidney disease status.

Variable		Total (N = 2940)	Patients without DKD (N = 1470)	Patients with DKD (N = 1470)	P-value
Age (years)		62.0 ± 10.3	62.0 ± 10.0	62.0 ± 10.5	–
Duration of Diabetes (years)		9 (4-15)	9 (4-15)	10 (5-15)	–
Gender	Men (N, %)	1276 (43.4%)	672 (45.7%)	604 (41.1%)	0.013
	Women (N, %)	1664 (56.6%)	798 (54.3%)	866 (58.9%)	
Hypertension (N, %)		1496 (50.9%)	663 (45.1%)	833 (56.7%)	<0.001
SBP (mmHg)		133.1 ± 19.4	131.8 ± 19.9	134.5 ± 18.8	<0.001
DBP (mmHg)		78.8 ± 10.3	78.3 ± 10.5	79.3 ± 10.1	0.015
WC (cm)		97.4 ± 10.4	95.8 ± 10.3	99.0 ± 10.3	<0.001
BMI (kg/m^2^)		28.6 ± 4.6	28.1 ± 4.5	29.1 ± 4.7	<0.001
FBS (mg/dL)		169.6 ± 64.7	168.7 ± 66.2	170.5 ± 63.1	0.455
2hpp (mg/dL)		237.8 ± 93.6	233.4 ± 90.4	242.1 ± 96.6	0.012
HbA1c (%)		7.96 ± 1.74	7.87 ± 1.74	8.04 ± 1.74	0.010
Creatinine (mg/dL)		1.06 ± 0.26	0.94 ± 0.16	1.19 ± 0.28	<0.001
eGFR (ml/min/1.73m^2^)		71.5 ± 18.4	80.3 ± 13.5	62.6 ± 18.5	<0.001
Triglycerides (mg/dL)		149 (107-207)	140 (101-190.25)	160 (113-226)	<0.001
Total Cholesterol (mg/dL)		177.7 ± 45.8	175.9 ± 44.0	179.6 ± 47.6	0.031
LDL-C (mg/dL)		99.7 ± 35.3	99.2 ± 34.1	100.2 ± 36.4	0.447
HDL-C (mg/dL)		44.9 ± 11.3	45.7 ± 11.7	44.2 ± 10.8	<0.001
Non-HDL-C (mg/dL)		132.8 ± 44.0	130.2 ± 42.2	135.4 ± 45.6	0.001
Medication					
Dyslipidemia drug N (%)	Atorvastatin	2500 (85.0%)	1326 (90.2%)	1174 (79.9%)	<0.001
	Rosuvastatin	356 (12.1%)	119 (8.1%)	237 (16.1%)	
	Fibrates	84 (2.9%)	25 (1.7%)	59 (4.0%)	
Antihyperglycemic agents N (%)	Multiple Drug Therapy	875 (29.8%)	272 (18.5%)	603 (41.0%)	<0.001
	Insulin	14 (0.5%)	2 (0.1%)	12 (0.8%)	
	Metformin monotherapy	1870 (63.6%)	1143 (77.8%)	727 (49.5%)	
	Any sulfonylurea	181 (6.2%)	53 (3.6%)	128 (8.7%)	

Data are presented as mean ± SD, median (Q1, Q3), or number (%).

DKD: diabetic kidney disease; SBP: systolic blood pressure; DBP: diastolic blood pressure; WC: waist circumference; BMI: body mass index; FBS: fasting blood sugar; 2hpp: 2-hour postprandial blood glucose; HbA1c: hemoglobin A1c; eGFR: estimated glomerular filtration rate; LDL-C: low-density lipoprotein cholesterol; HDL-C: high-density lipoprotein cholesterol; Non-HDL-C: non-high-density lipoprotein cholesterol.

**Table 2:** Lipid indices by diabetic kidney disease status in the study population.

Lipid index	Total	Patients without DKD	Patients with DKD	P-value
AC	3.1 ± 1.3	3.0 ± 1.2	3.2 ± 1.3	<0.001
TG/HDL-C Ratio	3.4 (2.3-5.1)	3.2 (2.2-4.6)	3.7 (2.5-5.6)	<0.001
AIP	1.2 (0.8-1.6)	1.1 (0.8-1.5)	1.3 (0.9-1.7)	<0.001
LCI	55740.2 (29170.2-103223.4)	51177.7 (27295.4-92766.6)	62138.7 (31441.0-114054.4)	<0.001

Data are presented as mean ± SD or median (Q1, Q3).

DKD: diabetic kidney disease; AC: atherogenic coefficient; TG: triglycerides; HDL-C: high-density lipoprotein cholesterol; AIP: atherogenic index of plasma; LCI: lipoprotein combine index.

### Restricted Cubic Spline analysis of lipid indices and DKD

**Fig 1:**
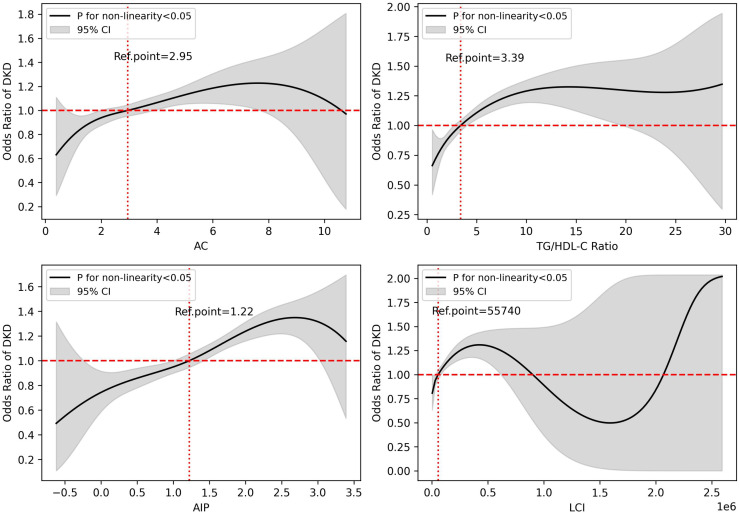
Association between lipid indices and DKD. Restricted cubic spline functions, with 4 knots placed at the 5^th^, 35^th^, 65^th^, and 95^th^ percentiles of each lipid index distribution, illustrate the ORs for having DKD in comparison to the median value of each index (reference point, OR=1). The curves demonstrate non-linear associations between lipid indices and DKD, with increasing ORs of having DKD as each index rises. DKD: diabetic kidney disease; OR: odds ratio; AC: atherogenic coefficient; TG: triglycerides; HDL-C: high-density lipoprotein cholesterol; AIP: atherogenic index of plasma; LCI: lipoprotein combine index.

Fig 1 illustrates the RCS model. The median value of each index was used as the reference, and the OR was set to one at that value. The reference values for the mentioned curves were 2.95 for AC, 3.39 for TG/HDL-C ratio, 1.22 for AIP, and 55740 for LCI. A nonlinear association was detected between all lipid indices and the presence of DKD (Fig 1). The ORs and 95% CIs of the RCS model with four knots (5th, 35th, 65th, and 95th percentiles for each index distribution) were as follows:

AC:
  - At the 5th percentile (1.47): OR = 0.81 (95% CI: 0.72–0.91)
  - At the 35th percentile (2.52): OR = 0.96 (95% CI: 0.89–1.02)
  - At the 65th percentile (3.38): OR = 1.01 (95% CI: 0.94–1.08)
  - At the 95th percentile (5.41): OR = 1.17 (95% CI: 1.10–1.32)
TG/HDL-C ratio:
  - At the 5th percentile (1.39): OR = 0.82 (95% CI: 0.71–0.94)
  - At the 35th percentile (2.70): OR = 0.95 (95% CI: 0.89–1.02)
  - At the 65th percentile (4.24): OR = 1.08 (95% CI: 1.02–1.14)
  - At the 95th percentile (9.53): OR = 1.31 (95% CI: 1.17–1.43)
AIP:
  - At the 5th percentile (0.33): OR = 0.80 (95% CI: 0.69–0.91)
  - At the 35th percentile (0.99): OR = 0.97 (95% CI: 0.90–1.04)
  - At the 65th percentile (1.44): OR = 1.04 (95% CI: 0.97–1.11)
  - At the 95th percentile (2.25): OR = 1.33 (95% CI: 1.21–1.43)
LCI:
  - At the 5th percentile (13254.79): OR = 0.83 (95% CI: 0.72–0.95)
  - At the 35th percentile (38471.47): OR = 0.99 (95% CI: 0.93–1.05)
  - At the 65th percentile (79445.19): OR = 1.00 (95% CI: 0.95–1.06)
  - At the 95th percentile (254755.59): OR = 1.33 (95% CI: 1.21–1.45)

### ROC curves for lipid indices in predicting DKD

**Fig 2:**
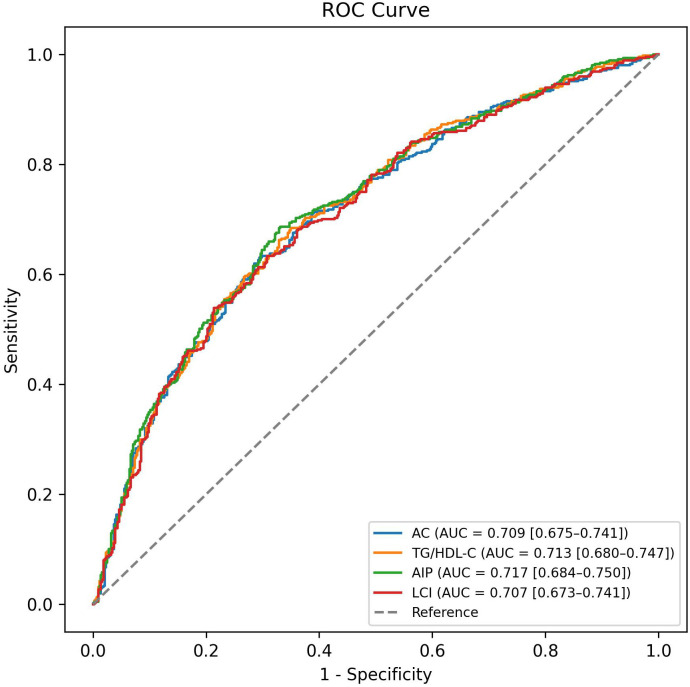
ROC curves showing the AUC for each lipid index in identifying DKD; adjusted for sex, hypertension, dyslipidemia drug, body mass index, antihyperglycemic agents, and HbA1c. The AIP demonstrated the pest performance, with an AUC of 0.717, a cutoff value of 0.84, and an accuracy of 68%. ROC: Receiver operating characteristic; AUC: area under the curve; DKD: diabetic kidney disease; AC: atherogenic coefficient; TG: triglycerides; HDL-C: high-density lipoprotein cholesterol; AIP: atherogenic index of plasma; LCI: lipoprotein combine index.

Fig 2 displays the Receiver Operating Characteristic Curves (ROCs) and area under the curve (AUC) values for various lipid indices in predicting DKD, with adjustments made for sex, hypertension, dyslipidemia drug, BMI, antihyperglycemic agents, and HbA1c. The AUCs for these indices were all above 0.707, with the highest value of 0.717 (95% confidence intervals [CI]: 0.684–0.750) for AIP. A cutoff value of 0.84 for AIP showed 68% sensitivity and 67% specificity in predicting DKD (P-value<0.001). Following AIP, the next highest AUC values belonged to TG/HDL-C ratio (AUC = 0.713, 95% CI: 0.680–0.747), AC (AUC = 0.709, 95% CI: 0.675–0.741), and LCI (AUC = 0.707, 95% CI: 0.673–0.741). Additional details on cutoff values, sensitivity, specificity, and p-values can be found in Table 3.

**Table 3:** AUC, sensitivity, and specificity at optimized cutoff points for lipid indices.

Lipid index	Cutoff	AUC (95% CI) ^a^	Sensitivity	Specificity	Accuracy	P-value
AC	2.45	0.709 (0.675-0.741)	63%	71%	67%	<0.001
TG/HDL-C Ratio	1.53	0.713 (0.680-0.747)	68%	66%	67%	<0.001
AIP	0.84	0.717 (0.684-0.750)	68%	67%	68%	<0.001
LCI	13036.2	0.707 (0.673-0.741)	54%	79%	66%	<0.001

^a^: Adjusted for sex, hypertension, dyslipidemia drug, body mass index, antihyperglycemic agents, and HbA1c.

AUC: area under the curve; CI: confidence interval; AC: atherogenic coefficient; TG: triglycerides; HDL-C: high-density lipoprotein cholesterol; AIP: atherogenic index of plasma; LCI: lipoprotein combine index.

### Association between quartiles of lipid indices and DKD

Each indicator was evaluated both as continuous variable and in quartiles, with the first quartile using as the reference category (OR=1) (Table 4). All indices had a significant association with DKD in the adjusted multivariable logistic regression analysis. In the case of AIP and TG/HDL-C ratio, quartiles 2, 3, and 4 had ORs (95% CI) of 1.27 (1.02–1.57), 1.33 (1.07–1.65), and 2.00 (1.59–2.50), respectively for the presence of DKD compared to the first quartile. Table 4 demonstrates that quartile 2 (OR=1.32; 95%CI: 1.06–1.64) and quartile 4 (OR=1.47; 95%CI: 1.18–1.84) of AC had significantly greater odds of DKD compared to the reference quartile. Additionally, the third (OR=1.29; 95%CI: 1.04–1.60) and fourth (OR=1.57; 95%CI: 1.25–1.96) quartiles of LCI showed 29% and 57% elevated odds of having DKD compared to the first quartile, respectively (Table 4).

**Table 4:** Association of lipid indices with diabetic kidney disease.

Lipid indices		OR of DKD (95% CI)	
		Unadjusted	Adjusted ^a^
AC	Continuous (Per one unit)	1.13 (1.07–1.20)	1.13 (1.06–1.20)
	Q1	Ref	–
	Q2	1.29 (1.05-1.58)	1.32 (1.06-1.64)
	Q3	1.23 (1.01-1.51)	1.20 (0.97-1.50)
	Q4	1.50 (1.22-1.84)	1.47 (1.18-1.84)
TG/HDL-C Ratio	Continuous (Per one unit)	1.09 (1.06–1.12)	1.08 (1.05–1.11)
	Q1	Ref	–
	Q2	1.27 (1.04-1.57)	1.27 (1.02-1.57)
	Q3	1.41 (1.14-1.73)	1.33 (1.07-1.65)
	Q4	2.16 (1.76-2.66)	2.00 (1.59-2.50)
AIP	Continuous (Per one unit)	1.61 (1.42–1.83)	1.51 (1.32–1.74)
	Q1	Ref	–
	Q2	1.27 (1.04-1.57)	1.27 (1.02-1.57)
	Q3	1.41 (1.14-1.73)	1.33 (1.07-1.65)
	Q4	2.16 (1.76-2.66)	2.00 (1.59-2.50)
LCI	Continuous (Per 10000 unit)	1.02 (1.01–1.03)	1.02 (1.01–1.02)
	Q1	Ref	–
	Q2	1.22 (0.99-1.50)	1.24 (0.99-1.54)
	Q3	1.33 (1.08-1.63)	1.29 (1.04-1.60)
	Q4	1.71 (1.39-2.10)	1.57 (1.25-1.96)

^a^: Adjusted for sex, hypertension, dyslipidemia drug, body mass index, antihyperglycemic agents, and HbA1c.

DKD: diabetic kidney disease; OR: odds ratio; CI: confidence interval; AC: atherogenic coefficient; TG: triglycerides; HDL-C: high-density lipoprotein cholesterol; AIP: atherogenic index of plasma; LCI: lipoprotein combine index; Q1: first quartile; Q2: second quartile; Q3: third quartile; Q4: fourth quartile.

### Stratified analyses by DKD components

Stratified analyses were performed based on eGFR and uACR cutoffs (S1 File). Patients were categorized into two groups by eGFR (<60 and ≥60 mL/min/1.73m^2^) and uACR levels (≥30 and <30 mg/g) and were compared. The TG/HDL-C ratio and AIP levels were significantly higher in patients with eGFR < 60 mL/min/1.73m^2^ compared to those with eGFR ≥ 60 mL/min/1.73m^2^ (p-value = 0.004), while no significant differences were detected for other lipid indices. Nonetheless, all lipid indices were significantly higher in individuals with uACR ≥ 30 mg/g compared to those with uACR < 30 mg/g (all p-values <0.001). RCS model showed non-linear associations between lipid indices and the odds of having eGFR < 60 mL/min/1.73m^2^. However, only AIP exhibited a statistically significant relationship. In contrast, all lipid indices showed significant non-linear associations with uACR ≥ 30 mg/g, with increasing levels of each index related to higher odds. In ROC curve analysis, AIP demonstrated the best performance for identifying eGFR < 60 mL/min/1.73m^2^, with an AUC (95% CI) of 0.676 (0.637–0.712), a cutoff value of 0.84, sensitivity of 60%, and specificity of 68%. For detecting uACR ≥ 30 mg/g, LCI showed the highest performance (cutoff = 44996.6, AUC = 0.741 [0.701–0.783], sensitivity = 68%, specificity = 73%). In multivariable logistic regression, each unit increase in AIP was associated with 41% higher odds of having eGFR < 60 mL/min/1.73m^2^. Additionally, compared to the first quartile, the ORs (95% CIs) of having eGFR < 60 mL/min/1.73m^2^ for the second, third, and fourth quartiles of AIP were 1.23 (0.98–1.56), 1.34 (1.05–1.69), and 1.79 (1.41–2.27), respectively. The third and fourth quartiles of the TG/HDL-C ratio were significantly associated with higher odds of having eGFR < 60 mL/min/1.73m^2^. Other lipids indices did not significantly correlate with eGFR < 60 mL/min/1.73m^2^. Concerning uACR ≥ 30 mg/g, higher quartiles of all lipid indices were significantly associated with increased odds compared to the first quartile, especially in the fourth quartile. Each unit increase in AIP was related to 24% higher odds of having uACR ≥ 30 mg/g. For LCI, the ORs (95% CIs) of having uACR ≥ 30 mg/g for the second, third, and fourth quartiles were 1.46 (1.09–1.96), 1.35 (1.01–1.80), and 1.89 (1.42–2.52), respectively, compared to the first quartile.

## Discussion

This study demonstrated that several novel lipid indices, including the TG/HDL-C ratio, AIP, AC, and LCI, were independently associated with DKD. All these indices were significantly higher in patients with DKD compared to those without DKD. Furthermore, the analysis revealed that both increasing continuous values and higher quartiles of lipid indices were associated with increased odds of DKD.

This study showed a significant non-linear association between TG/HDL-C ratio and DKD. The association between the TG/HDL-C ratio and DKD remains controversial. Some studies have identified this ratio as a marker of microvascular complications in T2D [36,37]. A cohort study in Italy found that patients with higher TG/HDL-C ratios had twice the risk of developing nephropathy and retinopathy over five years [36]. Similarly, a Japanese longitudinal study reported an association between elevated TG/HDL-C ratios and greater eGFR decline, and higher CKD incidence in patients with diabetes [38]. In contrast, other research has found no significant link between TG/HDL-C and diabetic nephropathy [39]. Mechanistically, elevated TG levels promote ceramide and nitric oxide production, which causes beta-cell death and insulin resistance [40], while reduced HDL-C impairs cholesterol efflux, leading to cholesterol accumulation within β-cells, further compromising their function [40]. Studies have shown that the TG/HDL-C ratio could reflect insulin resistance and atherogenic dyslipidemia [36,37,41,42]. Insulin resistance can impair renal function through glomerular hyperfiltration, oxidative stress, lipid toxicity, and glomerular sclerosis [43,44]. In addition, TG/HDL-C is correlated with LDL particle size and visceral adiposity, both of which may exacerbate inflammation, oxidative stress, and atherosclerosis, further contributing to renal dysfunction and DKD [36,45,46]. Therefore, the observed association between the TG/HDL-C ratio and DKD is biologically plausible. However, due to conflicting findings in the literature, further studies are needed to confirm this relationship.

The current study identified a significant non-linear association between AIP and DKD, supporting its potential as a marker for microvascular complications in T2D. AIP reflects lipoprotein particle size and is correlated with elevated TG, VLDL-C, and reduced HDL-C, which are indicative of atherogenic dyslipidemia [12,13]. Elevated AIP has been linked to increased risks of atherosclerosis, coronary heart disease, and vascular sclerosis [12,47]. Studies have suggested that AIP predicts the occurrence and severity of microvascular complications, including nephropathy and retinopathy [12,13,48]. However, not all studies support this association. Li et al. in a study involving 2523 patients with T2D found no significant difference in diabetic nephropathy incidence across AIP tertiles [49]. Elevated AIP reflects lipid metabolism disorders, lipid accumulation, insulin resistance, and chronic inflammation [50]. These factors lead to lipotoxicity, oxidative stress, mitochondrial dysfunction, glomerular hyperfiltration, endothelial damage, and pro-inflammatory adipokine release, resulting in glomerular and tubular injury, fibrosis, and kidney dysfunction [12,43,44,50,51].

There is limited research on the associations between AC and LCI indices and the DKD. In this study, both of these indices were independently associated with DKD. Elevated levels of these indices in DKD are likely driven by the lipid abnormalities characteristic of diabetes, including elevated TG, TC, and LDL-C, and decreased HDL-C levels [15,52]. Insulin resistance and metabolic dysregulation in diabetes further exacerbate lipid abnormalities, amplifying lipid indices values and increasing susceptibility to DKD [41,53]. The presence of small, dense LDL-C particles and oxidized LDL exacerbates endothelial dysfunction, oxidative stress, and inflammation, contributing to glomerular damage and vascular atherosclerosis [53]. AC, a marker reflecting the balance between non-HDL-C and HDL-C, is positively correlated with all atherogenic lipoprotein components in T2D and may serve as a surrogate for Apolipoprotein B measurement, making it a valuable marker for identifying atherogenic dyslipidemia and its contribution to DKD [13]. While LCI has been previously linked to conditions such as atherosclerosis, CVD, and non-alcoholic fatty liver disease (NAFLD) [54], this study is the first to report its independent association with DKD, highlighting its potential relevance in understanding lipid-driven mechanisms in DKD progression.

In this study, the lipid indices demonstrated relatively high predictive ability for DKD, with all AUC values exceeding 0.707. Among these, the AIP and TG/HDL-C ratio had the highest AUCs at 0.717 and 0.713, respectively, confirming their good discriminative ability for identifying DKD. The AIP and TG/HDL-C ratio also exhibited the highest sensitivity (68%), while LCI had the highest specificity (79%), highlighting the complementary diagnostic strengths of these indices. Compared to earlier research, the predictive accuracy observed in this study was higher. Tam et al. reported an AUC of 0.580 for TG/HDL-C in predicting diabetic nephropathy, with sensitivity and specificity of approximately 58% and 52%, respectively [39]. Mu et al. demonstrated an AUC of 0.652 for TG/HDL-C in predicting DKD [55]. Additionally, Yan et al. found an AUC of 0.592 for AIP, with sensitivity and specificity of about 58% and 54%, respectively [11]. The enhanced performance of lipid indices in this study may be due to improved methodologies or a more representative population sample. Given their low cost, ease of measurement, and non-invasive nature, novel lipid indices offer practical tools for early DKD detection, providing significant value in clinical settings and aiding timely intervention. These indices can be regularly evaluated through standard lipid profile testing, making them suitable for extensive clinical application.

It is worth noting that the cross-sectional design of the current study prevents us from drawing conclusions about temporal or causal relationships. Although this study revealed significant associations between various lipid indices and DKD, the results should be interpreted with caution, as it is not possible to distinguish whether lipid indices contribute to the progression of DKD or are its consequences. Future prospective cohort studies are required to explore the causative relationships and to confirm whether these lipid indices can predict the onset or progression of DKD.

### Strength and limitations

This study included a large group of individuals with T2D to examine the relationship between lipid indices and DKD. To address the limitations associated with observational nature of this study, we employed various methodological approaches. Initially, participants were matched based on age and duration of diabetes, two known risk factors for DKD, to mitigate potential confounding [56]. Additionally, we adjusted the analysis for several factors, including sex, hypertension, BMI, medications, and HbA1c, to reduce any residual confounding. However, some limitations of this study should be acknowledged. Firstly, this study was conducted at a single center, which may limit the generalizability of its findings. Secondly, as mentioned, the cross-sectional design prevents establishing a causal relationship between lipid indices and DKD. Additionally, despite adjustments for several confounding factors, unmeasured variables such as genetic predispositions, dietary habits, and psychosocial influences might have impacted the results. Addressing these limitations will require further research through multicenter, prospective cohort studies.

## Conclusion

This study demonstrated that lipid indices, including AIP, TG/HDL-C ratio, AC, and LCI, are independently associated with DKD. These indices offer a valuable, non-invasive tool for assessing the presence of DKD in individuals with T2D. Integrating these lipid indices into routine clinical practice could enhance early detection and more effective diabetes management, potentially reducing the burden of DKD.

## Supporting information

S1 File Supporting Information. Supplementary methods, tables, and figures presenting analyses of lipid indices and their associations with DKD, eGFR, and uACR.
(PDF)

S2 File Data. The dataset used for the analyses presented in the study.
(XLSX)
