# Supplementary material for: Can lipid indices aid in predicting diabetic kidney disease? Findings from a cross-sectional, matched case-control study
Source: PLoS One. 2025 Oct 7;20(10):e0331756. doi: 10.1371/journal.pone.0331756 (PMC12503270; doi:10.1371/journal.pone.0331756)
Supplement: S1 File — Supplementary methods, tables, and figures presenting analyses of lipid indices and their associations with DKD, eGFR, and uACR. (PDF) [file pone.0331756.s001.pdf]

Supporting Information

Can lipid indices aid in predicting diabetic kidney disease? Findings from a cross-sectional, matched case-control study

Amirhossein Yadegar, Fatemeh Mohammadi, Fatemeh Heydarzadeh, Kiavash Mokhtarpour, Sepideh Yadegar, Rana Hashemi, Seyed Ali Nabipoorashrafi, Soghra Rabizadeh, Alireza Esteghamati, Manouchehr Nakhjavani

Supporting Methods (CRI-I and CRI-II formula) ………… Page 2

S1 Table (Comparison of CRI-I and CRI-II levels between different groups) ………… Page 2

S2 Table (Odds ratios of the RCS model for CRI-I and CRI-II) ………… Page 2

S3 Table (Results of ROC curve analysis for CRI-I and CRI-II) ………… Page 2

S4 Table (Association of CRI-I and CRI-II with diabetic kidney disease) ………… Page 3

S1 Fig (RCS model for CRI-I and CRI-II) ………… Page 4

S2 Fig (ROC curve analysis for CRI-I and CRI-II) ………… Page 5

S5 Table (Baseline characteristics according to eGFR and uACR cutoffs) ………… Page 6

S6 Table (Comparison of lipid indices according to eGFR and uACR cutoffs) ………… Page 7

S7 Table (Odds ratios from the RCS model for the eGFR <60 or uACR ≥30) ………… Page 8

S8 Table (ROC curve analysis for identifying eGFR <60 or uACR ≥30) ………… Page 9

S9 Table (Association of lipids indices with eGFR <60 or uACR ≥30) ………… Page 10

S3 Fig (RCS model for eGFR <60) ………… Page 12

S4 Fig (RCS model for uACR ≥30) ………… Page 13

S5 Fig (ROC curve analysis for eGFR <60 and uACR ≥30) ………… Page 14

Supporting Methods:

CRI-I and CRI-II were excluded from the primary analyses due to high multicollinearity with other lipid indices. However, their results are included here.

Formula: CRI-I = TC/HDL-C; CRI-II = LDL-C/HDL-C

S1 Table. Comparison of CRI-I and CRI-II levels between different groups in the study population

Lipid index	Total	Patients without DKD	Patients with DKD	P-value
CRI-I	4.1±1.3	4.0±1.2	4.2±1.3	<0.001
CRI-II	2.3±0.9	2.3±0.9	2.4±0.9	0.015

Data are presented as mean ± SD

DKD: diabetic kidney disease; CRI-I: Castelli risk index-I; CRI- II: Castelli risk index- II

S2 Table. The odds ratios and 95% CIs from the RCS model with four knots (5th, 35th, 65th, and 95th percentiles) for CRI-I and CRI-II in relation to the DKD

Lipid index	Knots											
	5th percentile			35th percentile			65th percentile			95th percentile		
	Value	OR	95% CI	Value	OR	95% CI	Value	OR	95% CI	Value	OR	95% CI
CRI-I	2.47	0.81	0.72-0.91	3.52	0.96	0.89-1.02	4.38	1.01	0.94-1.08	6.41	1.21	1.10-1.32
CRI-II	1.09	0.98	0.92-1.05	1.87	1.00	0.93-1.07	2.54	1.05	0.94-1.15	4.00	1.17	1.05-1.28

DKD: diabetic kidney disease; OR: odds ratio; CI: confidence interval; RCS: restricted cubic spline; CRI-I: Castelli risk index-I; CRI- II: Castelli risk index- II

S3 Table. AUC, sensitivity, and specificity of CRI-I and CRI-II for identifying DKD

Lipid index	Cutoff	AUC (95% CI) [a]	Sensitivity	Specificity	Accuracy	P-value
CRI-I	5.14	0.709 (0.675-0.741)	63%	71%	67%	<0.001
CRI-II	2.22	0.705 (0.669-0.738)	57%	75%	66%	<0.001

[a]: Adjusted for sex, hypertension, dyslipidemia drug, body mass index, antihyperglycemic agents, and HbA1c

AUC: area under the curve; CI: confidence interval; CRI-I: Castelli risk index-I; CRI- II: Castelli risk index- II; DKD: diabetic kidney disease

S4 Table. Association of CRI-I and CRI-II with diabetic kidney disease

Lipid indices		OR of DKD (95% CI)	
		Unadjusted	Adjusted [a]
CRI-I	Continuous (Per one unit)	1.13 (1.07–1.20)	1.13 (1.06–1.20)
	Q1	Ref	-
	Q2	1.29 (1.05-1.58)	1.32 (1.06-1.64)
	Q3	1.23 (1.01-1.51)	1.20 (0.97-1.50)
	Q4	1.50 (1.22-1.84)	1.47 (1.18-1.84)
CRI-II	Continuous (Per one unit)	1.10 (1.02–1.19)	1.11 (1.02–1.20)
	Q1	Ref	-
	Q2	0.97 (0.79-1.19)	0.99 (0.80-1.23)
	Q3	1.07 (0.87-1.32)	1.12 (0.90-1.39)
	Q4	1.19 (0.97-1.46)	1.19 (0.95-1.48)

[a]: Adjusted for sex, hypertension, dyslipidemia drug, body mass index, antihyperglycemic agents, and HbA1c

DKD: diabetic kidney disease; OR: odds ratio; CI: confidence interval; CRI-I: Castelli risk index-I; CRI- II: Castelli risk index- II; Q1: first quartile; Q2: second quartile; Q3: third quartile; Q4: fourth quartile

S1 Fig. Association between CRI-I and CRI-II and DKD. The restricted cubic spline function, using 4 knots derived from the distribution of each index, illustrates the ORs for having DKD in comparison to the median value of each index (reference point)

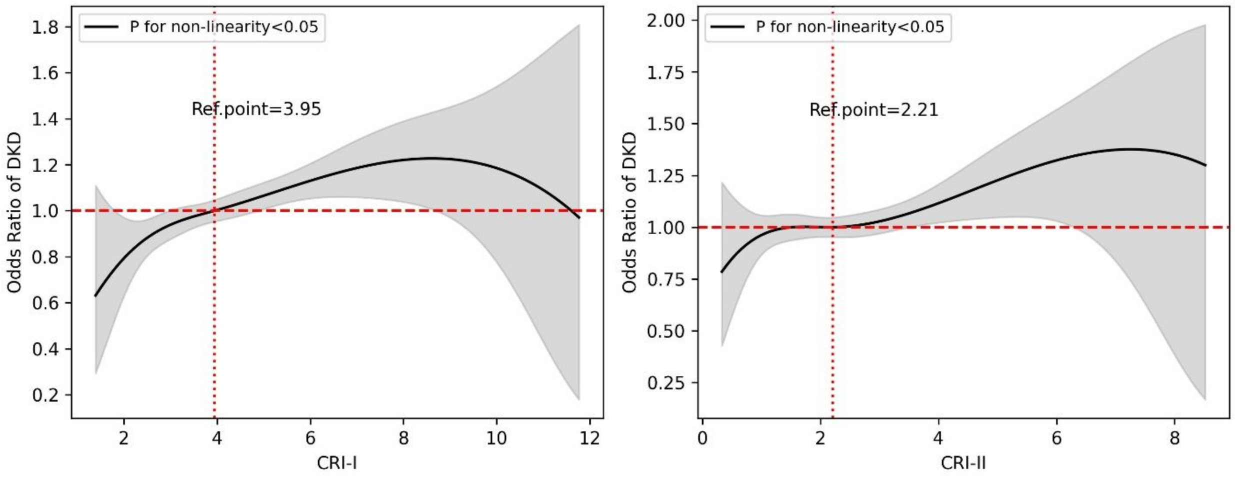

DKD: diabetic kidney disease; OR: odds ratio; RCS: restricted cubic spline; CRI-I: Castelli risk index-I; CRI-II: Castelli risk index- II

S2 Fig. ROC curves showing the AUCs of CRI-I and CRI-II in identifying DKD; adjusted for sex, hypertension, dyslipidemia drug, body mass index, antihyperglycemic agents, and HbA1c

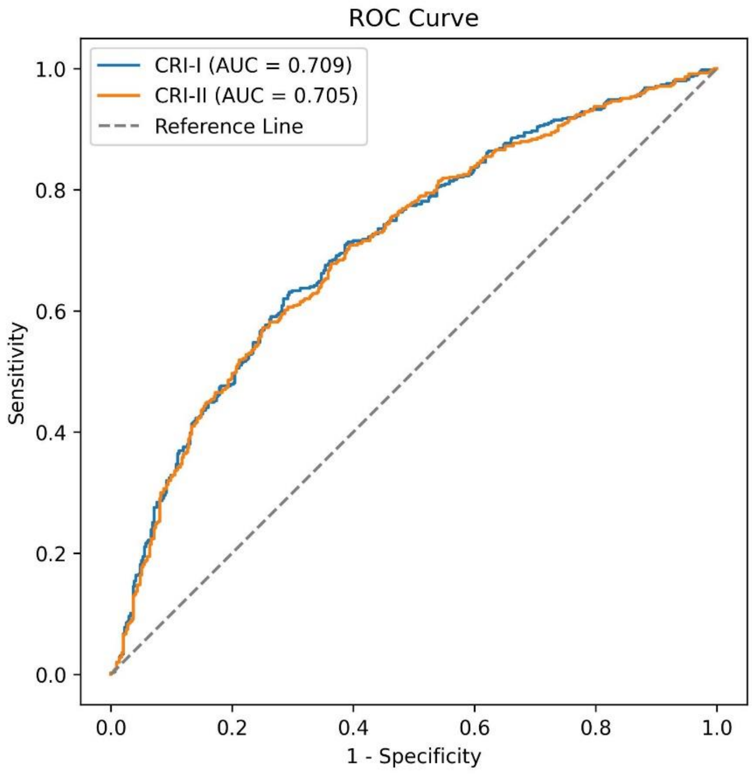

ROC: Receiver operating characteristic; AUC: area under the curve; DKD: diabetic kidney disease; CRI-I: Castelli risk index-I; CRI- II: Castelli risk index- II

S5 Table. Baseline characteristics of the study population according to eGFR and uACR cutoffs

Variable		eGFR			uACR		
		≥60 mL/min/1.73m^2 (N=2009)	<60 mL/min/1.73m^2 (N=931)	P-value	<30 mg/g (N=2269)	≥30 mg/g (N=671)	P-value
Age (years)		60.7±10.4	64.9±9.2	<0.001	63.1±9.9	58.3±10.7	<0.001
Duration of Diabetes (years)		9 (4-15)	10 (5-15)	0.354	8 (4-15)	10 (6-18)	<0.001
Gender	Men (N, %)	967 (48.1%)	309 (33.2%)	<0.001	928 (40.9%)	348 (51.9%)	<0.001
	Women (N, %)	1042 (51.9%)	622 (66.8%)		1341 (59.1%)	323 (48.1%)	
Hypertension (N, %)		932 (46.4%)	564 (60.6%)	<0.001	1127 (49.7%)	369 (55.0%)	0.016
SBP (mmHg)		132.2±19.4	135.1±19.3	<0.001	132.7±19.5	134.5±18.9	0.039
DBP (mmHg)		78.9±10.3	78.5±10.2	0.275	78.3±10.4	80.6±9.8	<0.001
WC (cm)		96.9±10.4	98.5±10.3	<0.001	96.6±10.3	100.0±10.4	<0.001
BMI (kg/m^2)		28.4±4.5	29.1±4.7	<0.001	28.4±4.5	29.2±4.7	<0.001
FBS (mg/dL)		172.7±65.9	162.8±61.5	<0.001	165.8±64.1	182.3±65.0	<0.001
2hpp (mg/dL)		242.3±93.6	227.9±92.9	<0.001	230.1±91.4	263.7±96.3	<0.001
HbA1c (%)		8.02±1.75	7.82±1.71	0.004	7.82±1.72	8.41±1.75	<0.001
Creatinine (mg/dL)		0.94±0.16	1.32±0.25	<0.001	1.07±0.26	1.04±0.25	0.007
eGFR (ml/min/1.73m^2)		81.0±13.5	50.9±7.7	<0.001	70.0±18.3	76.4±18.1	<0.001
Triglycerides (mg/dL)		146 (105-204)	155 (111-216)	0.004	144 (105-198)	172 (117-241)	<0.001
Total Cholesterol (mg/dL)		179.3±45.2	174.3±47.0	0.007	174.9±45.1	187.3±47.1	<0.001
LDL-C (mg/dL)		101.3±34.8	96.2±36.1	<0.001	97.9±34.9	105.5±36.0	<0.001
HDL-C (mg/dL)		45.1±11.3	44.6±11.4	0.243	45.3±11.6	43.7±10.2	0.001
Non-HDL-C (mg/dL)		134.2±43.7	129.7±44.6	0.012	129.6±42.9	143.6±46.0	<0.001
Medication							
Dyslipidemia drug N (%)	Atorvastatin	1707 (85.0%)	793 (85.2%)	0.374	2011 (88.6%)	489 (72.9%)	<0.001
	Rosuvastatin	239 (11.9%)	117 (12.6%)		216 (9.5%)	140 (20.9%)	
	Fibrates	63 (3.1%)	21 (2.2%)		42 (1.9%)	42 (6.2%)	
Antihyperglycemic agents N (%)	Multiple Drug Therapy	525 (26.1%)	350 (37.6%)	<0.001	556 (24.5%)	319 (47.5%)	<0.001
	Insulin	5 (0.2%)	9 (1.0%)		11 (0.5%)	3 (0.4%)	
	Metformin monotherapy	1358 (67.6%)	512 (55.0%)		1600 (70.5%)	270 (40.2%)	
	Any sulfonylurea	121 (6.0%)	60 (6.4%)		102 (4.5%)	79 (11.8%)	

Data are presented as mean ± SD, median (Q1, Q3), or number (%)

eGFR: estimated glomerular filtration rate; uACR: urine albumin-creatinine ratio; SBP: systolic blood pressure; DBP: diastolic blood pressure; WC: waist circumference; BMI: body mass index; FBS: fasting blood sugar; 2hpp: 2-hour postprandial blood glucose; HbA1c: hemoglobin A1c; LDL-C: low-density lipoprotein cholesterol; HDL-C: high-density lipoprotein cholesterol; Non-HDL-C: non-high-density lipoprotein cholesterol.

S6 Table. Comparison of lipid indices between different groups according to eGFR and uACR cutoffs

Lipid index	eGFR			uACR		
	≥60 mL/min/1.73m^2	<60 mL/min/1.73m^2	P-value	<30 mg/g	≥30 mg/g	P-value
CRI-I	4.1±1.3	4.1±1.2	0.115	4.03±1.22	4.46±1.37	<0.001
CRI-II	2.3±0.9	2.2±0.9	0.055	2.26±0.89	2.52±1.00	<0.001
AC	3.1±1.3	3.1±1.2	0.115	3.03±1.22	3.46±1.37	<0.001
TG/HDL-C Ratio	3.3 (2.3-5.0)	3.6 (2.4-5.3)	0.004	3.3 (2.2-4.8)	4.0 (2.5-6.2)	<0.001
AIP	1.2 (0.8-1.6)	1.3 (0.9-1.7)	0.004	1.2 (0.8-1.6)	1.4 (0.9-1.8)	<0.001
LCI	56114.7 (29354.1-104845.2)	55111.9 (28511.3-96228.0)	0.420	51394.5 (27579.6-92592.9)	71237.3 (36996.7-134013.0)	<0.001

Data are presented as mean ± SD or median (Q1, Q3)

eGFR: estimated glomerular filtration rate; uACR: urine albumin-creatinine ratio; CRI-I: Castelli risk index-I; CRI- II: Castelli risk index- II; AC: atherogenic coefficient; TG: triglycerides; HDL-C: high-density lipoprotein cholesterol; AIP: atherogenic index of plasma; LCI: lipoprotein combine index.

S7 Table. The odds ratios and 95% CIs from the RCS model for lipid indices in relation to the presence of eGFR <60 mL/min/1.73m² or uACR ≥30 mg/g

eGFR<60 mL/min/1.73m²	Knots							
	5th percentile		35th percentile		65th percentile		95th percentile	
	Value	OR (95% CI)	Value	OR (95% CI)	Value	OR (95% CI)	Value	OR (95% CI)
CRI-I	2.47	1.00 (0.86-1.15)	3.52	1.02 (0.93-1.13)	4.38	0.95 (0.86-1.04)	6.41	0.90 (0.74-1.07)
CRI-II	1.09	1.25 (1.09-1.42)	1.87	1.06 (0.95-1.16)	2.54	0.94 (0.85-1.03)	4.00	0.95 (0.79-1.14)
AC	1.47	1.00 (0.86-1.15)	2.52	1.02 (0.93-1.13)	3.38	0.95 (0.86-1.04)	5.41	0.90 (0.74-1.07)
TG/HDL-C Ratio	1.39	0.87 (0.72-1.02)	2.70	0.92 (0.84-1.01)	4.24	1.07 (0.99-1.15)	9.53	0.96 (0.78-1.15)
AIP	0.33	0.83 (0.69-0.99)	0.99	0.94 (0.84-1.04)	1.44	1.04 (0.95-1.15)	2.25	1.00 (0.84-1.17)
LCI	13254.8	0.97 (0.82-1.14)	38471.5	1.02 (0.94-1.10)	79445.2	0.95 (0.88-1.03)	254755.6	1.05 (0.90-1.23)
uACR≥30 mg/g	Knots							
	5th percentile		35th percentile		65th percentile		95th percentile	
	Value	OR (95% CI)	Value	OR (95% CI)	Value	OR (95% CI)	Value	OR (95% CI)
CRI-I	2.47	0.49 (0.37-0.64)	3.52	0.99 (0.87-1.12)	4.38	0.95 (0.85-1.07)	6.41	1.67 (1.42-1.93)
CRI-II	1.09	0.74 (0.59-0.92)	1.87	0.92 (0.81-1.05)	2.54	0.99 (0.88-1.10)	4.00	1.46 (1.22-1.71)
AC	1.47	0.49 (0.37-0.64)	2.52	0.99 (0.87-1.12)	3.38	0.95 (0.85-1.07)	5.41	1.67 (1.42-1.93)
TG/HDL-C Ratio	1.39	0.77 (0.58-1.00)	2.70	0.97 (0.85-1.10)	4.24	1.20 (1.08-1.33)	9.53	1.75 (1.43-2.10)
AIP	0.33	0.77 (0.60-0.99)	0.99	0.93 (0.81-1.07)	1.44	1.11 (0.98-1.25)	2.25	1.83 (1.56-2.12)
LCI	13254.8	0.56 (0.40-0.77)	38471.5	0.98 (0.87-1.09)	79445.2	1.10 (0.99-1.21)	254755.6	1.80 (1.57-2.05)

CI: confidence interval; OR: odds ratio; RCS: restricted cubic spline; eGFR: estimated glomerular filtration rate; uACR: urine albumin-creatinine ratio; CRI-I: Castelli risk index-I; CRI- II: Castelli risk index- II; AC: atherogenic coefficient; TG: triglycerides; HDL-C: high-density lipoprotein cholesterol; AIP: atherogenic index of plasma; LCI: lipoprotein combine index.

S8 Table. AUC, sensitivity, and specificity of lipid indices for identifying eGFR <60 mL/min/1.73m² or uACR ≥30 mg/g

eGFR<60 mL/min/1.73m^2						
Lipid index	Cutoff	AUC (95% CI) [a]	Sensitivity	Specificity	Accuracy	P-value
CRI-I	3.69	0.662 (0.624-0.699)	49%	77%	68%	<0.001
CRI-II	3.70	0.662 (0.623-0.700)	47%	78%	68%	<0.001
AC	4.65	0.662 (0.624-0.699)	49%	77%	68%	<0.001
TG/HDL-C Ratio	4.49	0.673 (0.634-0.710)	59%	69%	66%	<0.001
AIP	0.84	0.676 (0.637-0.712)	60%	68%	66%	<0.001
LCI	45682.9	0.663 (0.624-0.700)	49%	77%	68%	<0.001
uACR≥30 mg/g						
Lipid index	Cutoff	AUC (95% CI) [b]	Sensitivity	Specificity	Accuracy	P-value
CRI-I	4.63	0.740 (0.699-0.781)	62%	78%	74%	<0.001
CRI-II	0.85	0.739 (0.699-0.781)	61%	80%	75%	<0.001
AC	1.67	0.740 (0.699-0.781)	62%	78%	74%	<0.001
TG/HDL-C Ratio	3.55	0.739 (0.699-0.781)	64%	77%	74%	<0.001
AIP	1.96	0.738 (0.697-0.779)	65%	76%	73%	<0.001
LCI	44996.6	0.741 (0.701-0.783)	68%	73%	72%	<0.001

[a]: Adjusted for age, sex, hypertension, body mass index, fasting blood sugar, HbA1c, antihyperglycemic agents

[b]: Adjusted for age, duration of diabetes, sex, hypertension, body mass index, fasting blood sugar, HbA1c, antihyperglycemic agents, and dyslipidemia drug

AUC: area under the curve; CI: confidence interval; eGFR: estimated glomerular filtration rate; uACR: urine albumin-creatinine ratio; CRI-I: Castelli risk index-I; CRI- II: Castelli risk index- II; AC: atherogenic coefficient; TG: triglycerides; HDL-C: high-density lipoprotein cholesterol; AIP: atherogenic index of plasma; LCI: lipoprotein combine index.

S9 Table. Association of lipids indices with eGFR <60 mL/min/1.73m² or uACR ≥30 mg/g

Lipid indices		OR of eGFR<60 mL/min/1.73m^2 (95% CI)		OR of uACR≥30 mg/g (95% CI)	
		Unadjusted	Adjusted [a]	Unadjusted	Adjusted [b]
CRI-I	Continuous (Per one unit)	0.95 (0.89–1.01)	1.03 (0.96–1.10)	1.29 (1.21–1.37)	1.15 (1.07–1.24)
	Q1	Ref	-	Ref	-
	Q2	1.02 (0.82–1.27)	1.15 (0.92–1.44)	1.57 (1.20–2.04)	1.39 (1.04–1.84)
	Q3	0.89 (0.71–1.10)	1.04 (0.82–1.31)	1.64 (1.26–2.14)	1.28 (0.96–1.70)
	Q4	0.86 (0.69–1.07)	1.12 (0.88–1.42)	2.38 (1.85–3.07)	1.64 (1.24–2.18)
CRI-II	Continuous (Per one unit)	0.89 (0.81–0.96)	0.97 (0.89–1.06)	1.33 (1.22–1.46)	1.20 (1.08–1.33)
	Q1	Ref	-	Ref	-
	Q2	0.81 (0.66–1.01)	0.89 (0.71–1.12)	1.39 (1.07–1.80)	1.26 (0.95–1.68)
	Q3	0.78 (0.63–0.97)	0.91 (0.73–1.15)	1.62 (1.25–2.09)	1.43 (1.08–1.89)
	Q4	0.71 (0.57–0.89)	0.88 (0.70–1.11)	2.10 (1.64–2.70)	1.60 (1.21–2.11)
AC	Continuous (Per one unit)	0.95 (0.89–1.01)	1.03 (0.96–1.10)	1.29 (1.21–1.37)	1.15 (1.07–1.24)
	Q1	Ref	-	Ref	-
	Q2	1.02 (0.82–1.27)	1.15 (0.92–1.44)	1.57 (1.20–2.04)	1.39 (1.04–1.84)
	Q3	0.89 (0.71–1.10)	1.04 (0.82–1.31)	1.64 (1.26–2.14)	1.28 (0.96–1.70)
	Q4	0.86 (0.69–1.07)	1.12 (0.88–1.42)	2.38 (1.85–3.07)	1.64 (1.24–2.18)
TG/HDL-C Ratio	Continuous (Per one unit)	1.03 (1.00–1.05)	1.06 (1.04–1.09)	1.09 (1.06–1.12)	1.02 (0.99–1.05)
	Q1	Ref	-	Ref	-
	Q2	1.26 (1.01–1.57)	1.25 (0.99–1.58)	1.14 (0.87–1.49)	1.15 (0.86–1.53)
	Q3	1.25 (1.00–1.56)	1.35 (1.06–1.71)	1.49 (1.15–1.93)	1.26 (0.95–1.67)
	Q4	1.41 (1.13–1.77)	1.77 (1.40–2.26)	2.28 (1.78–2.91)	1.48 (1.12–1.95)
AIP	Continuous (Per one unit)	1.19 (1.05–1.36)	1.41 (1.21–1.63)	1.74 (1.50–2.02)	1.24 (1.05–1.47)
	Q1	Ref	-	Ref	-
	Q2	1.24 (1.00–1.55)	1.23 (0.98–1.56)	1.11 (0.85–1.44)	1.11 (0.83–1.47)
	Q3	1.24 (0.99–1.55)	1.34 (1.05–1.69)	1.48 (1.15–1.92)	1.26 (0.95–1.66)
	Q4	1.42 (1.14–1.77)	1.79 (1.41–2.27)	2.27 (1.77–2.90)	1.46 (1.10–1.92)
LCI	Continuous (Per 10000 unit)	1.00 (0.99–1.01)	1.00 (1.00–1.01)	1.03 (1.02–1.03)	1.01 (1.00–1.02)
	Q1	Ref	-	Ref	-
	Q2	0.96 (0.77–1.20)	1.03 (0.82–1.29)	1.50 (1.15–1.96)	1.46 (1.09–1.96)
	Q3	1.05 (0.85–1.31)	1.13 (0.90–1.42)	1.55 (1.19–2.02)	1.35 (1.01–1.80)
	Q4	0.89 (0.71–1.11)	1.07 (0.84–1.35)	2.75 (2.13–3.54)	1.89 (1.42–2.52)

[a]: Adjusted for age, sex, hypertension, body mass index, fasting blood sugar, HbA1c, antihyperglycemic agents

[b]: Adjusted for age, duration of diabetes, sex, hypertension, body mass index, fasting blood sugar, HbA1c, antihyperglycemic agents, and dyslipidemia drug

OR: odds ratio; CI: confidence interval; eGFR: estimated glomerular filtration rate; uACR: urine albumin-creatinine ratio; CRI-I: Castelli risk index-I; CRI- II: Castelli risk index- II; AC: atherogenic coefficient; TG: triglycerides; HDL-C: high-density lipoprotein cholesterol; AIP: atherogenic index of plasma; LCI: lipoprotein combine index; Q1: first quartile; Q2: second quartile; Q3: third quartile; Q4: fourth quartile

S3 Fig. Association between lipids indices and eGFR <60 mL/min/1.73m². The restricted cubic spline function, using 4 knots derived from the distribution of each index, illustrates the ORs for having eGFR <60 mL/min/1.73m² in comparison to the median value of each index (reference point)

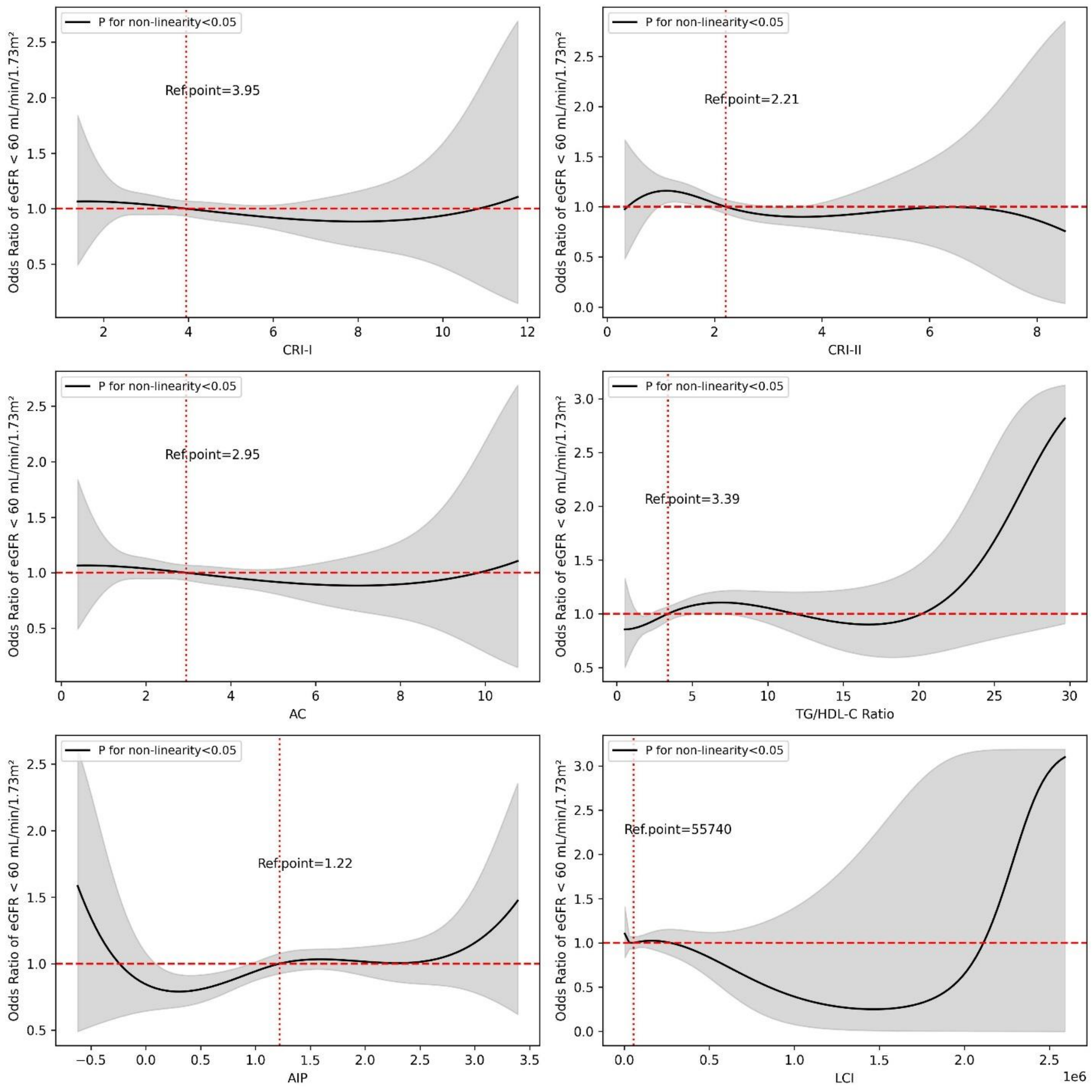

OR: odds ratio; CI: confidence interval; eGFR: estimated glomerular filtration rate; CRI-I: Castelli risk index-I; CRI- II: Castelli risk index- II; AC: atherogenic coefficient; TG: triglycerides; HDL-C: high-density lipoprotein cholesterol; AIP: atherogenic index of plasma; LCI: lipoprotein combine index

S4 Fig. Association between lipids indices and uACR ≥30 mg/g. The restricted cubic spline function, using 4 knots derived from the distribution of each index, illustrates the ORs for having uACR ≥30 mg/g in comparison to the median value of each index (reference point)

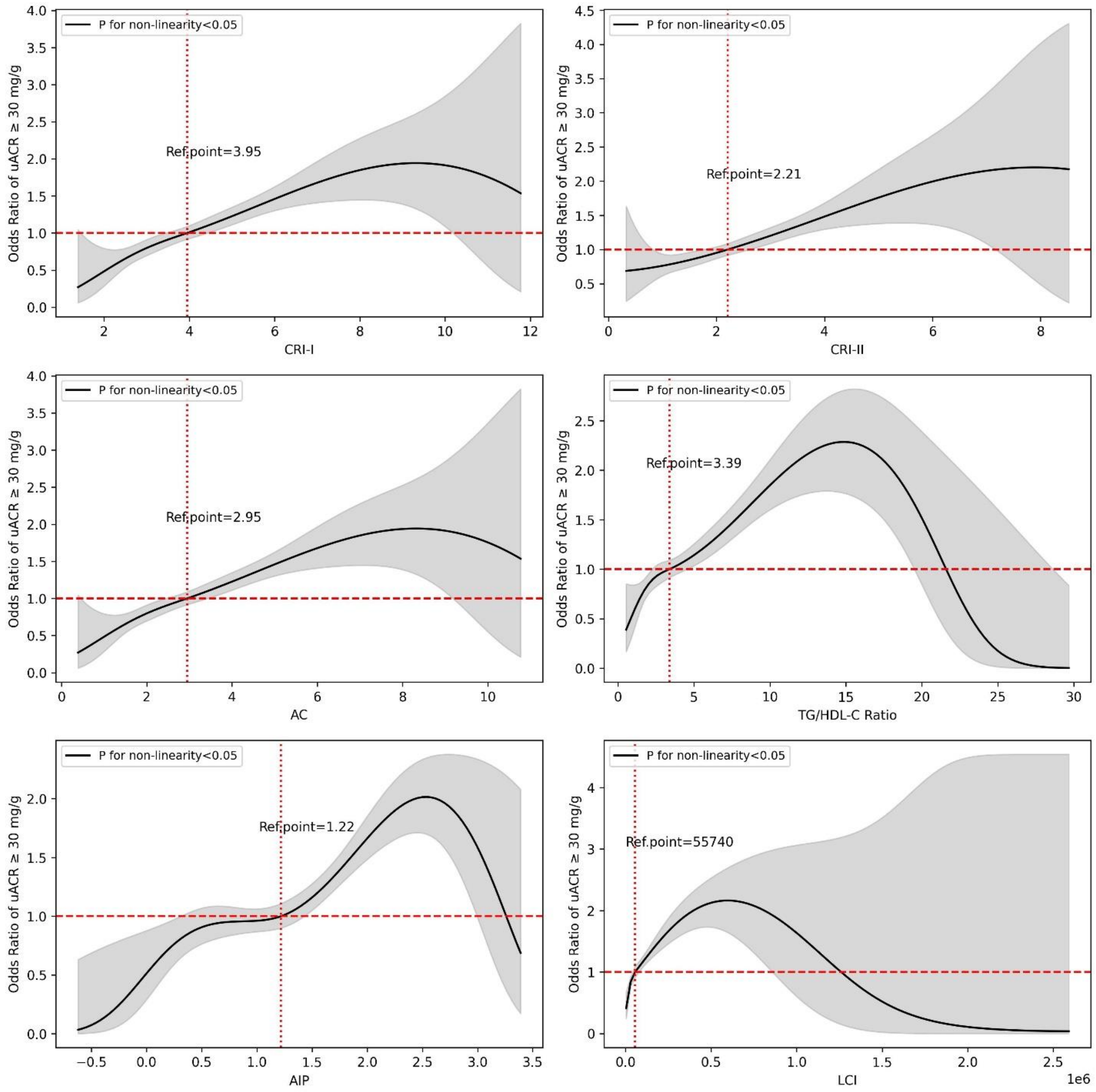

OR: odds ratio; CI: confidence interval; uACR: urine albumin-creatinine ratio; CRI-I: Castelli risk index-I; CRI- II: Castelli risk index- II; AC: atherogenic coefficient; TG: triglycerides; HDL-C: high-density lipoprotein cholesterol; AIP: atherogenic index of plasma; LCI: lipoprotein combine index

S5 Fig. ROC curves showing the AUCs of lipid indices in identifying eGFR <60 mL/min/1.73m² or uACR ≥30 mg/g

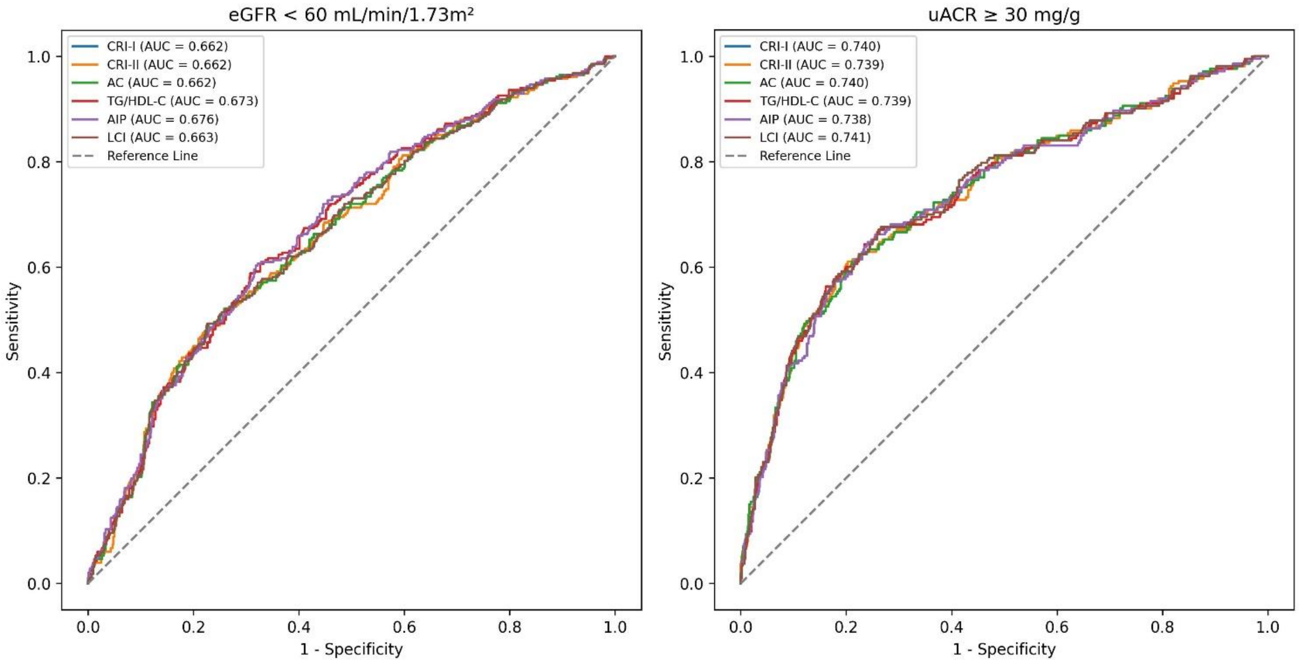

eGFR curve: Adjusted for age, sex, hypertension, body mass index, fasting blood sugar, HbA1c, antihyperglycemic agents

uACR curve: Adjusted for age, duration of diabetes, sex, hypertension, body mass index, fasting blood sugar, HbA1c, antihyperglycemic agents, and dyslipidemia drug

ROC: Receiver operating characteristic; AUC: area under the curve; eGFR: estimated glomerular filtration rate; uACR: urine albumin-creatinine ratio; CRI-I: Castelli risk index-I; CRI- II: Castelli risk index- II; AC: atherogenic coefficient; TG: triglycerides; HDL-C: high-density lipoprotein cholesterol; AIP: atherogenic index of plasma; LCI: lipoprotein combine index
